# Very short answer questions: a viable alternative to multiple choice questions

**DOI:** 10.1186/s12909-020-02057-w

**Published:** 2020-05-06

**Authors:** Thomas Puthiaparampil, Md Mizanur Rahman

**Affiliations:** 1 Department of Medicine, Faculty of Medicine and Health Sciences, Universiti Malaysia Sarawak, UNIMAS, 94300 Kota Samarahan, Malaysia; 2 Department of Community Medicine and Public Health, Faculty of Medicine and Health Sciences, Universiti Malaysia Sarawak, UNIMAS, 94300 Kota Samarahan, Malaysia

**Keywords:** Very short answer questions, Multiple choice questions, Best answer questions

## Abstract

**Background:**

Multiple choice questions, used in medical school assessments for decades, have many drawbacks such as hard to construct, allow guessing, encourage test-wiseness, promote rote learning, provide no opportunity for examinees to express ideas, and do not provide information about strengths and weakness of candidates. Directly asked, directly answered questions like Very Short Answer Questions (VSAQ) are considered a better alternative with several advantages.

**Objectives:**

This study aims to compare student performance in MCQ and VSAQ and obtain feedback.

from the stakeholders.

**Methods:**

Conduct multiple true-false, one best answer, and VSAQ tests in two batches of medical students, compare their scores and psychometric indices of the tests and seek opinion from students and academics regarding these assessment methods.

**Results:**

Multiple true-false and best answer test scores showed skewed results and low psychometric performance compared to better psychometrics and more balanced student performance in VSAQ tests. The stakeholders’ opinions were significantly in favour of VSAQ.

**Conclusion and recommendation:**

This study concludes that VSAQ is a viable alternative to multiple-choice question tests, and it is widely accepted by medical students and academics in the medical faculty.

## Background

Multiple True/False (MTF) and One Best Answer Questions (BAQ) are widely employed by the medical faculties by virtue of their advantages of instant machine scoring, freedom from examiner bias, and dependable reliability [1–4]. In this article, ‘MCQ’ is used to refer to both these instruments of assessment. The reliability of a test is higher when the subject coverage is wider [5, 6]. Reliability refers to test reproducibility with similar results when used for different batches, but it does not ensure validity [2, 3]. MCQ tests seem to sacrifice validity for reliability [3]. A primary purpose of any assessment is to let students know what is important to learn [6]. Assessments are supposed also to enable feedback to students and direct the teaching strategies [1, 7–10], and this is applicable especially to formative assessments. MCQ tests do not provide information, which would enable feedback, as they do not require students to construct the answers [1]. It is an established fact that assessments drive the learning style [1, 6, 10, 11]. MCQ is blamed for promoting rote learning, guessing, test-wiseness, and turning students into data banks [2, 5, 7, 12–14]. MCQ tends to test trivia [2] and they are not able to test complex issues [3, 7]. MCQ does not provide any opportunity for students to express their understanding [15]. What is required in a medical school is learning that leads to the formation of competent doctors, which MCQ tests are incapable of assessing fully [3]. Constructing good MCQs with appropriate difficulty and discriminating ability needs expertise and experience, which are often lacking [6, 15, 16]. Discrimination index (DISi) in MCQ may be deceptive as good students may find them more difficult to answer, as the questions may be the authors’ opinions and not well-known facts [2, 15]. True-false MCQ may even have a negative effect on students, attributable to the false statements, which they might take home as true [10]. It appears that the demerits of MCQ are numerous and overwhelming, and there is strong backing for alternative instruments like directly asked, directly answered questions.

## Objectives of the study

This study aims to test directly asked, directly answered questions like very short answer questions (VSAQ) in medical students, compare their performance with MCQ tests and seek opinions from participating students and academics of the Faculty of Medicine and Health Sciences, Universiti Malaysia Sarawak, regarding MTF, BAQ and VSAQ.

## Methods

### Study participants and their selection

This was a cross-sectional study conducted in the Faculty of Medicine and Health Sciences, Universiti Malaysia Sarawak (UNIMAS), a public university in Malaysia. The study was conducted for a period of six months in 2019. The year-3 batch of medical students is divided into four groups and year-5 batch into three groups and each group undergoes the clinical postings in rotations. Whole groups of 37 year-3 and 39 year-5 students, who were undergoing medical postings during this study period, were formally recruited for the VSAQ tests. Students’ participation was voluntary and they signed the consent form. The VSAQ tests were not part of any assessment. The MTF and BAQ tests, of which student scores and item analysis were used in this study, were part of end of posting examinations. MTF and BAQ questions used in the faculty examinations are written by the concerned lecturers. These students, whose MTF and BAQ results were used, did not fill the feedback questionnaire, while study participating students filled the feedback questionnaire immediately after the VSAQ tests.

### Instrument and data collection

VSAQ papers were prepared by converting the MTF and BAQ items used for the end of posting examinations of the groups of students who completed their medical postings just before the groups taking VSAQ tests underwent medical posting. This meant that the questions were testing the same knowledge using different instruments in different student groups of the same standard. Year-5 VSAQ test consisted of 32 items, marks per item ranged from 1 to 4 coming to a total of 100. Each item had vignettes followed by 1–4 questions, each question carrying one mark. Year-3 VSAQ consisted of 53 items, simpler and directly asked without vignettes, marks per item ranged from 1 to 5 coming to a total of 95, which was converted to 100. Test time allowed was 1 h for both groups. All the answers were handwritten in spaces provided after each question in the printed question papers. The number of words allowed in the answers was not specified. The VSAQ answer scripts were manually scored based on predetermined model answers. Answers falling outside the model answers were considered while marking. The two questionnaires, one for the students and another for the academics, were prepared by the authors. Although the items in the questionnaires were not validated statistically, the content analysis was done by the authors and validated by a language expert. The academics’ views on MTF, BAQ, VSAQ were obtained using a Google Form questionnaire with 17 items.

### Data analysis

The item analysis of MTF and BAQ was done by Smartscan Optical Mark Reader while scoring student scripts, as it is always done in the faculty. The marks of VSAQ tests were entered into a Microsoft Excel Worksheet and their item analysis performed manually. The formulae used for item analysis are shown in the Appendix. The data were described with descriptive statistics in terms of means and standard deviation for continuous variables and frequencies and percentages for categorical variables. An independent sample t-test was done to compare the scores of MTF, BAQ, and VSAQ. To determine any correlation between the two assessments (continuous data), the bivariate Pearson correlation coefficient was calculated. The difference between MTF vs. VSAQ and BAQ vs. VSAQ, (student’s performance as qualitative data) were tested by Pearson’s Chi-square test of independence. Item-wise student feedback on VSAQ, MTF, and BAQ were tested between year-5 and year-3 students using Pearson’s Chi-square test of independence. A *p*-value of ≤0.05 was considered as statistically significant. IBM SPSS version 22.0 was used for statistical analysis. The academics’ views on MTF, BAQ and VSAQ were analysed manually and described as percentages.

## Results

### Time taken for answering tests

The VSAQ test with 53 questions and 14-item feedback were completed by year-3 students in 30–45 min. The year-5 students also completed the VSAQ test with 32 items and 14-item feedback in less than 50 min. The time allotted for both the MTF tests of 20 items was 50 min and for the BAQ test of 15 items 45 min, as practiced in the faculty.

### Student performance in tests

**Fig. 1:**
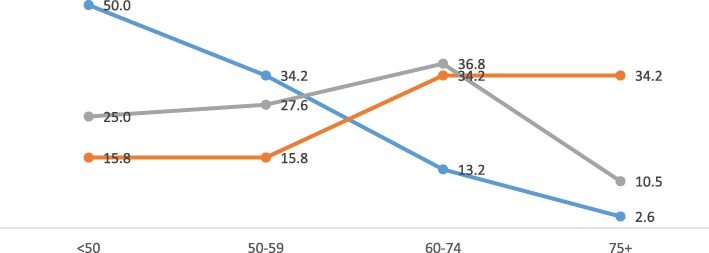
Overall student performance in MTF, BAQ and VSAQ *p*-value reached from Pearson’s Chi-square test of independence MTF vs. VSAQ (*p* < 0.05) and BAQ vs. VSAQ (p < 0.05) Percentage of students (y-axis) and students’ score grades (x-axis) Legend: MTF (blue), BAQ (Orange) and VSAQ (ash) The scores of year-3 students (37) and year-5 students (39) were added together for MTF, BAQ and VSAQ for this graph. The scores are distributed into 4 grades: < 50 = fail, 50–59 = bare pass, 60–74 = good pass and 75+ = distinction

It was observed that the student performance in MTF, BAQ and VSAQ was significantly higher in year-5 group compared to year-3 (*p* < 0.01). The test-wise analysis revealed a strong correlation between MTF and VSAQ performance both in year-3 and year-5 tests (*p* < 0.001). However, the relationship between BAQ and VSAQ performance was found to be weakly positive (*p* < 0.01) (Table 1). The overall performance in MTF showed a low trend with 50% failures in contrast to BAQ, which showed an upward trend with 34.2% of students scoring distinction marks. At the same time, student performance in VASQ showed a desirable and balanced distribution with a decline in distinction scorers compared to BAQ (Fig. 1).

**Table 1:** Year-3 and year-5 Students’ scores in the 6 Tests

Test	Y3	Q	Mean (SD)	Min, Max	Y5	Q	Mean (SD)	Min, Max	§p-value
MTF	37	20	45.5 (9.4)	25, 54	39	20	55.0 (11.1)	29, 76	p < 0.001***
BAQ	37	15	54.9 (12.8)	31.7, 82.3	39	15	76.9 (11.6)	53.3, 100	p < 0.001***
VSAQ	37	53	54.1 (12.4)	33.7, 77.9	39	32	62.9 (11.2)	38, 91	p < 0.01**
VSAQ vs. MTF (r = 0.714***)					VSAQ vs. MTF (r = 0.632***)				
VSAQ vs. BAQ (r = 0.435**)					VSAQ vs. BAQ (r = 0.321*)				

*p < 0.05, **p < 0.01, ***p < 0.001

*§p-*value reached from independent sample t test

Y3 = year-3, Y5 = year-5 number of students, Q = number of questions, Min = minimum, Max = maximum, SD = standard deviation, MTF = multiple true-false, BAQ = best answer questions, VSAQ = very short answer questions

### Item analysis of tests

**Fig. 2:**
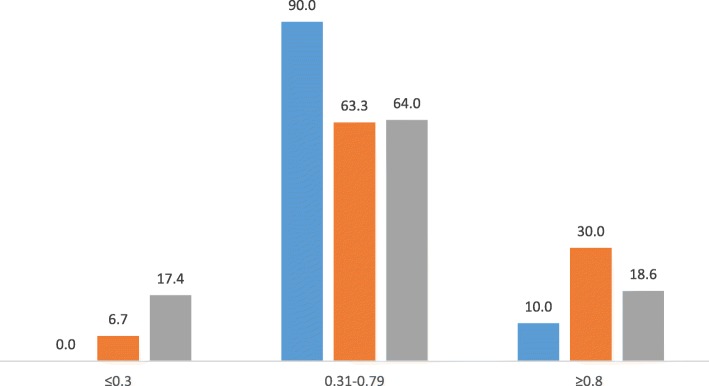
Percentage distribution of the difficulty index of 6 tests Percentage of questions (y-axis) and difficulty index categories (x-axis) Legend: MTF (blue), BAQ (orange) and VSAQ (ash) The item analysis values of year-3 tests and year-5 tests were added together for MTF, BAQ and VSAQ for this graph. The bars show percentages of items falling in 3 categories: ≤0.3 (too difficult), 0.31–0.79 (moderate difficulty), and ≥ 0.8 (easy) as per Ebel and Frisbe [17]

**Fig. 3:**
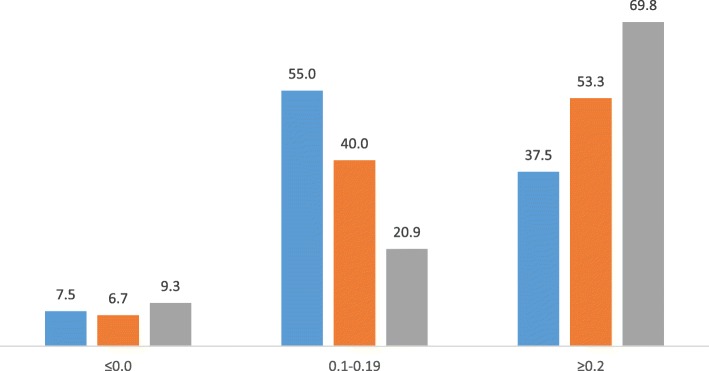
Percentage distribution of the discrimination index of MTF, BAQ and VSAQ Percentage of questions (y-axis) and discrimination index categories (x-axis) Legend: MTF (blue), BAQ (orange) and VSAQ (ash) The percentages of year-3 and year-5 test items (combined) falling in 3 discrimination index categories are shown. MTF (20 items), BAQ (4-option, 15 items) and VSAQ (54 items Y-3 and 32 items Y-5) are combined for this figure. Different groups of students of the same batch participated in the study. The VSAQ papers were prepared by converting the MTF and BAQ questions. The bars show percentages of items falling in the three categories: ≤0.0 = no or negative discrimination; 0.1–0.19 = fair and ≥ 0.2 good and excellent distribution combined. Item analysis of VSAQ was manually performed on Excel Worksheet using the ‘UNIMAS Formulae’ [18]

Most MTF items fell in the moderate difficulty category, BAQ showed a trend towards more easy items, while VSAQ showed a balanced distribution (Fig. 2). As for discrimination index, MTF showed a fair distribution, BAQ was higher than MTF, while about 70% VSAQ items compared to 37.5% MTF and 53% BAQ achieved a discrimination index of 0.2 and higher (Fig. 3).

### Students’ feedback on MTF, BAQ and VSAQ

Most of the students expressed opinions in favour of VSAQ. More than four-fifths (86.5%) of them opined that VSAQ is an efficient method of assessment of knowledge, and equal proportions of them opined that VSAQ provides an opportunity for students to express their ideas. About 80% in both groups were happy to see this new method of assessment, and 59.5 and 79.5% of them expressed interest to see more VSAQ tests. Overall the feedback showed a high acceptance of VSAQ (Table 2). There was a significant similarity in the opinions expressed by year-3 and year-5 students about MCQ tests. They did not consider MCQ tests any better in assessing knowledge compared to VSAQ. Over 62% (year-3) and 53% (year-5) students considered BAQ easy to answer, as they contained many distractors easy to exclude as the answer. A good number of students, more of them in year-5 group, had encountered confusing MTF and BAQ items in their examinations (Table 3).

**Table 2:** Students’ feedback on VSAQ

***Statements***	***Year-3 (n = 37)***			***Year-5 (n = 39)***			***p-value***
	***In favour***	***Neutral***	***Against***	***In favour***	***Neutral***	***Against***	
VSAQ is an efficient method of assessment of knowledge	86.5	13.5	0.0	89.7	10.3	00.0	p > 0.05
VSAQ can test more items compared to MTF and BAQ in the same amount of time	70.3	10.8	18.9	89.7	10.3	0.0	p < 0.05
VSAQ tests knowledge better as the students need to write the answers	62.2	24.3	13.5	79.5	7.7	12.8	p > 0.05
VSAQ provides an opportunity for students to express their ideas	86.5	10.8	2.7	89.7	7.7	2.6	p > 0.05
Happy to see a new assessment method	78.4	21.6	0.0	87.2	12.8	0.0	p > 0.05
I would like to see more VSAQ tests.	62.2	32.4	5.4	79.5	15.4	5.1	p > 0.05

*p < 0.05, **p < 0.01, ***p < 0.001

*p-*value reached from Pearson’s chi-square test

Pearson’s chi-square test of independence revealed no statistically significant difference between year-3 and year-5 students’ opinions (*p* > 0.05) except in the second question

**Table 3:** Students’ feedback on MTF and BAQ

***Statements***	***Year-3 (n = 37)***			***Year-5 (n = 39)***			***p-value***
	***Agree***	***Not sure***	***Disagree***	***Agree***	***Not sure***	***Disagree***	
MTF is more efficient than VSAQ for testing knowledge	16.2	64.9	18.9	15.4	46.2	38.5	p > 0.05
BAQ is more efficient than VSAQ in testing knowledge	29.7	40.5	29.8	15.4	41.0	43.6	p > 0.05
MTFs, which I encountered had been clear and unambiguous	29.7	29.7	40.6	28.2	15.4	56.4	p > 0.05
BAQs are easy, as most distractors are easy to exclude to reach the answer	62.2	10.8	27.0	53.8	20.5	25.7	p > 0.05
	***Very often***	***Sometimes***	***Seldom***	***Very often***	***Sometimes***	***Seldom***	
Did you encounter confusing items in MTF?	0.0	10.8	89.2	59.0	35.9	5.1	***p < 0.001***
Did you encounter confusing items in BAQ?	0.0	43.2	56.8	28.2	51.3	20.5	***p < 0.001***
	***Very confident***	***OK***	***Not confident***	***Very confident***	***OK***	***Not confident***	
How confident have you been while answering MTF?	0.0	73.0	27.0	53.8	20.5	25.7	***p < 0.001***

*p < 0.05, **p < 0.01, ***p < 0.001

*p-*value reached from Pearson’s chi-square test

#### Lecturers’ feedback on MTF, BAQ and VSAQ

1. More than 90% were involved in constructing MTF and BAQ, but less than 10% of them mentioned it was a pleasant experience.
2. More than 90% felt that writing BAQ was more difficult than writing MTF. Over 67% considered writing 3–4 plausible distractors a difficult task.
3. In MTF, 87% considered writing of false options more difficult than writing true options. Twenty per cent of them said they converted true options to false, and some said they just wrote false options without care, as they were false anyway.
4. About using item analysis to improve the questions, 60% mentioned they did not do it.
5. Hundred per cent respondents said they got ideas about the students’ strengths and weaknesses while marking directly asked, directly answered questions like Modified Essay Questions (MEQ) and Short Answer Questions (SAQ), and 40% of them said they do not get any such information from MTF and BAQ results. However, 97% believed such information was important to guide teaching.
6. About their openness to VSAQ, 60% answered ‘surely’, and 40% answered ‘maybe’.

## Discussion

Students scored poorly in MTF with high failure rates and very few high scorers. This is a common trend in our faculty, which is generally attributed to the negative marking scheme used. On the contrary, BAQ was skewed towards high scorers with very few poor performers, which is also a common trend in our faculty. The VSAQ scores showed a fair and balanced distribution, as would be expected in a reliable test. There was a strong positive correlation between VSAQ and MTF both in year-3 and year-5 tests (*p* < 0.001), while the correlation between VSAQ and BAQ in both tests were weakly positive (*p* < 0.01). This discrepancy was somewhat reflected in the item analysis of these tests. While most MTF items fell in the moderate difficulty category, BAQ items were more easy and VSAQ more balanced in distribution. Items with 0.2 or higher DISi were more in VSAQ compared to MTF and BAQ, while items with lower DISi were more in both MTF and BAQ tests. Our overall results showed fairer and balanced student performance and superior psychometric properties of VSAQ compared to MTF and BAQ. VSAQ items were constructed based on MTF and BAQ, therefore testing the same knowledge. But the former being answered by students in a shorter time is simply because MTF (2.5 min/item) and BAQ (3 min/item) are allotted unduly longer test times than required in our faculty.

Students’ opinions about MTF were generally unfavourable. Mention of many confusing items in the questions were notable. Negative opinions about MTF and BAQ were more evident in year-5 students’ feedback, which reflected their seniority and the number of tests they would have taken compared to year-3 students. Generally, students’ opinions were highly in favour of VSQ. Hift [19] proposed elimination of open-ended questions like MEQ in summative assessments, as context-rich well written best answer questions had higher reliability and validity than MEQ. But this author also supported open-ended questions for formative assessments or assessments for learning. Open-ended questions were favoured also by college students [1].

The validity and reliability of tests depend on how widely the subject is covered in the assessment [5, 6], but unduly prolonged tests would be counterproductive. Our study showed that wider coverage of topics was easier to achieve in VSAQ tests compared to MCQ, as claimed in literature [3, 20]. This is evident as both the groups of students could complete the VSAQ tests with larger number of questions in shorter time compared to MTF and BAQ. Test-wiseness and guessing, which are unavoidable in MCQ tests are of no concern in VSAQ. It has also been observed that students found false options harder than true ones to answer, and omission rates due to uncertainty are high in MTF [21]. The adverse effect of using false options was also highlighted by Wood [10]. BAQ claims higher-order assessment than MTF but can be easier to score because of the many non-functioning distractors most of them contain [22].

Assessments are of immense value in letting students know what is important to learn [6] and for providing feedback to students and directing the teaching [1, 7, 8, 15, 23]. VSAQ was seen to achieve these ends better than MCQ. Reading the VSAQ answers while marking provides the examiner’s insight into students’ strengths and deficiencies, which would help in providing feedback and modifying the teaching [7, 20]. MCQ tests do not reveal with certainty what the students know, how much they know, and whether they are capable of using the knowledge in real-life situations, as they do not require the students to construct the answers [1]. Directly answered questions like VSAQ is an alternative, which will reveal more of students’ competence. Factual knowledge, important for a doctor [15], was effectively tested by our VSAQ tests. Higher taxonomy testing is also said to be better achieved in VSAQ [1]. VSAQs are easier to construct [12]. The struggle of writing MCQ was reflected in our academics’ feedback. Most of them considered writing good MTF and 3 to 4 plausible distractors in BAQ a difficult task, just as described in the literature [22]. Our students’ feedback highlighted the issue of confusing items in MCQ, while no such opinions were expressed about VSAQ. The requirement to construct the answers would drive better learning, as preparation strategies would change [1, 3, 10]. The motivation for learning will be better when students are required to express their knowledge in the examination [1]. Direct questions let students express their ideas [1, 20], which is a desired purpose of assessments. MCQ can be considered an assessment of convenience far from real-life medical practice, while direct questions are considered more natural [2].

In the past, when essay questions were used, the onus of constructing the answers was on students. With the advent of MCQ, the roles reversed. Now, the teachers need to cover the syllabus in questions, while the students do not need to write a single word, but to choose the correct answers either knowingly, by guessing or using test-wiseness. In effect, a switch from MCQ to VSAQ will transfer the burden of writing all the answers (true, false and distractors) from the teachers back to the students. Constructing questions will still be an expert job, albeit less arduous than MCQ, as it won’t require to create false options and plausible distractors. The only disadvantage envisaged in adopting VSAQ is the time spent on manual scoring. The time spent on constructing MCQ can now be utilised for marking scripts with more rewarding benefits. VSAQ can be scored by anyone with the help of model answers [7, 12], and the possibility of computer technology [3] making the task easier in future is real. Our academics and students responded positively to a shift from MCQ to VSAQ. The demerits of MCQ are many, and there is strong backing for VSAQ, but the transition, as usual, is slow to come. An electronic VSA exam platform has been developed by the UK Medical Schools Council Assessment Alliance to complement their existing SBA platform, which is already widely used by medical schools throughout the UK [24]. We are encouraged by the finding by Sam et al. [25] that VSAQ format is capable of high reliability, validity, discrimination, and authenticity, while SBAQ format was associated with significant cueing.

### Limitations of the study

This study was limited to one institution. All the three tests were not administered on the same group of students of year-3 and year-5 but on different groups of the same batch undergoing different rotations of the same course. So it is only presumed that they were of the same standard. The feedback questionnaires were not formally validated statistically.

## Conclusion

VSAQ employ directly asked questions, which require students to answer briefly and directly with no scope of guessing or reaching the answer by elimination. A larger quantity of knowledge recall, understanding and application can be tested in a shorter period compared to MCQ. The information obtained by the teachers while scoring will help to modify the teaching and to give feedback to the students. Previously used MTF and BAQ items can be converted to VSAQ effectively. Our study showed more balanced student performance and better psychometric indices in VSAQ compared to MTF and BAQ. The students by and large preferred VSAQ to MTF and BAQ. Our academics also expressed the deficiencies of MCQ and showed openness to VSAQ. Last but not the least VSAQ using computers will cut the costs and the carbon footprint of the faculty drastically. In conclusion, VSAQ would be a viable alternative to MCQ with many plus points.

## Data Availability

The data will not be available publicly. The principal author keeps all the materials and data used in this study. However, the questionnaires for this could be available from the first author.

## Appendix

**Formulae used for calculating difficulty index (DIFi) and discrimination index (DISi) of Multiple True-False (MTF), Best Answer Questions (BAQ) and Very Short Answewr Questions (VSAQ).**

**DIFi of MTF and BAQ.**

Number of examinees getting the item correct ÷ total number of examinees.

**DISi of MTF and BAQ.**

(Number of examinees getting the item correct among the 27% high scorers – number of examinees getting the item correct among the low scorers) ÷ number of exminees in one cohort.

**DIFi of VSAQ.**

Total score obtained by the examinees in an item ÷ maximum score obtainable by all examinees in that item.

**DISi of VSAQ.**

(Total score obtained by 27% top scorers in an item – total score obtained by 27% low scorers in that item) ÷ maximum scores obtainable by 27% examinees for the same item.

## Abbreviations

BAQ: Best Answer Questions
DIFi: Difficulty index
DISi: Discrimination index
IRB: Institutional Review Board
MCQ: Multiple Choice Questions
MTF: Multiple true-false
SBA: Single Best Answer
SBAQ: Single Best Answer Questions
VASQ: Very Short Answer Questions
UNIMAS: Universiti Malaysia Sarawak
